# Triple Conductive Wiring by Electron Doping, Chelation Coating and Electrochemical Conversion in Fluffy Nb_2_O_5_ Anodes for Fast‐Charging Li‐Ion Batteries

**DOI:** 10.1002/advs.202202201

**Published:** 2022-07-07

**Authors:** Yongjian Zheng, Wujie Qiu, Lei Wang, Jianjun Liu, Shuangqiang Chen, Chilin Li

**Affiliations:** ^1^ CAS Key Laboratory of Materials for Energy Conversion Shanghai Institute of Ceramics Chinese Academy of Sciences Shanghai 201899 China; ^2^ Center of Materials Science and Optoelectronics Engineering University of Chinese Academy of Sciences Beijing 100049 China; ^3^ State Key Laboratory of High Performance Ceramics and Superfine Microstructure Shanghai Institute of Ceramics Chinese Academy of Sciences 585 He Shuo Road Shanghai 201899 China; ^4^ Department of Chemical Engineering School of Environmental and Chemical Engineering Shanghai University Shangda Road 99 Shanghai 200444 China

**Keywords:** conductive wiring, conversion reaction, fast‐charging performance, lithium ion batteries, T–Nb_2_O_5_

## Abstract

High‐rate anode material is the kernel of developing fast‐charging lithium ion batteries (LIBs). T–Nb_2_O_5_, well‐known for its “room and pillar” structure and bulk pseudocapacitive effect, is expected to enable the fast lithium (de)intercalation. But this property is still limited by the low electronic conductivity or insufficient wiring manner. Herein, a strategy of triple conductive wiring through electron doping, chelation coating, and electrochemical conversion inside the microsized porous spheres consisting of dendrite‐like T–Nb_2_O_5_ primary particles is proposed to achieve the fast‐charging and durable anodes for LIBs. The penetrative implanting of conformal carbon coating (derivative from polydopamine chelate) and NbO domains (induced by excess discharging) reinforces the global supply of electronically conductive wires, apart from those from Co/Mn heteroatom or O vacancy doping. The polydopamine etching on T–Nb_2_O_5_ spheres promotes their evolution into fluffy morphology with better electrolyte infiltration. The synergic electron and ion wiring at different scales endow the modified T–Nb_2_O_5_ anode with ultralong cycling life (143 mAh g^−1^ at 1 A g^−1^ after 8500 cycles) and high‐rate performance (144.1 mAh g^−1^ at 10.0 A g^−1^). The permeation of multiple electron wires also enables a high mass loading of T–Nb_2_O_5_ (4.5 mg cm^−2^) with a high areal capacity of 0.668 mAh cm^−2^ even after 150 cycles.

## Graphical abstract

A strategy of triple conductive wiring through electron doping, chelation coating, and electrochemical conversion inside the microsized porous spheres consisting of dendrite‐like T–Nb_2_O_5_ primary particles is proposed to achieve the fast‐charging and durable anodes. The synergic electron and ion wiring at different scales endows the modified T–Nb_2_O_5_ anode with ultralong cycling life and high‐rate performance.

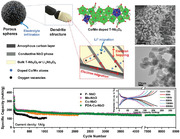

## 1 Introduction

Although the gradual proliferation of electric vehicles (EVs) is an unstoppable trend into our future, it remains a puzzle how to fill the remaining gap in performance between EV batteries and combustion engines. In particular, the criterion of US Advanced Battery Consumption (USABC) for high performance commercial batteries stipulates 80% state of charge achieved within 15 minutes during a regular recharge, which is still a challenge.^[^ ^1^, ^2^ ^]^ Realizing fast recharge can contribute to the market penetration and consumer acceptance of electrical vehicles. However, generally the practice of fast‐charging either sacrifices the energy density and mileage range by compromising the cell‐design metrics or risks the lifespan and safety of batteries otherwise. Current research efforts are thus attracted to the exploitation of high‐power electrode materials, which directly tackles the technical bottleneck.^[^ ^3^ ^]^

Graphite anode is the foundation for the commercialization of lithium‐ion batteries (LIBs) in 1991.^[^ ^4^ ^]^ Though graphite displays the relatively high specific capacity (372 mAh g^−1^) and highly reversible (de)intercalation performance, it is widely deemed to be the culprit that impedes the achievement of fast‐charging due to its low intercalation potential (≈0.1 V vs Li^+^/Li).^[^ ^5^ ^]^ As a promising alternative anode material, orthorhombic niobium pentoxide (T–Nb_2_O_5_) is well known for its intercalation‐based pseudocapacitive effect, enabling its high‐rate charge storage.^[^ ^6^ ^]^ Inside the distinct layered structure of T–Nb_2_O_5_ with “room and pillar” NbO_6_/NbO_7_ units, the covalent bonds (Nb–O–Nb) instead of weak van der Waals forces (usually between laminated carbon layers inside graphite) connect the adjacent niobium atom layers. This unique structure enables the formation of robust Li diffusion channels and prevents structural exfoliation as observed in graphite under ultrafast Li^+^ (de)intercalation.^[^ ^7^ ^]^ Furthermore, Li deposition is another well‐known limitation for graphite anode during high‐rate charging due to its low intercalation potential. This issue can also be circumvented in T–Nb_2_O_5_ considering its intercalation potential range (1.0–3.0 V).^[^ ^3^ ^]^ Moreover, micrometer‐scale Nb_2_O_5_ particles also display higher tap density than commercial graphite, Li_4_Ti_5_O_12_ and TiO_2_, promising an opportunity to further improve the cells’ volumetric energy density.^[^ ^8^ ^]^ It deserves to highlight that no requirement on complicate nanostructure design on Nb_2_O_5_ can mitigate the fracture of fragile solid electrolyte interface and avoid the unconscionable Li depletion. These prominent and luciferous prospects of T–Nb_2_O_5_ have attracted more and more researchers into the field, but it is also plagued by several intractable disadvantages (e.g., low electron conductivity) when compared with commercial graphite anode.

In general, stoichiometric Nb_2_O_5_ is identified as an electrical insulator (with a band gap of 3.2–4.0 eV) and it can be converted to n‐type semiconductor with the implantation of trace amount of oxygen vacancies.^[^ ^9^ ^]^ The intrinsically inferior electric conductivity of Nb_2_O_5_ (≈3 × 10^–6^ S cm^−1^) would trigger the particle pulverization and rapid capacity decay during the iterative charge/discharge process.^[^ ^10^, ^11^ ^]^ A variety of methods have been done to tackle this problem. For instance, a nitrogen‐doped amorphous carbon layer has been demonstrated to homogenize electron transport in monoclinic Nb_2_O_5_ (H–Nb_2_O_5_) so as to suppress inhomogeneous phase change, finally achieving excellent rate performance (≈120 mAh g^−1^ at 16.0 A g^−1^).^[^ ^12^ ^]^ Besides, Cu_2_Nb_34_O_87_, a compound based on Cu_2_O doped Nb_2_O_5_, exhibits enhanced mass and charge transport (3.5 × 10^–13^ cm^2^ s^−1^ for Li^+^ and 2.1 × 10^–5^ S cm^−1^ for electron). Analogously, WO_3_–Nb_2_O_5_ solid solution (Nb_14_W_3_O_44_) was found to facilitate the construction of interconnected 3D Li^+^ migration (10^–10^–10^–12^ cm^2^ s^−1^) tunnels in microsized particles.^[^ ^8^, ^13^ ^]^ Synergistic engineering of electron and ion transport pathways appears to be effective in promoting the rapid and reversible Li^+^ (de)intercalation in niobium‐based oxides. Exterior conductive carbon wiring and interior defect‐rich structuring synergistically endow Nb_2_O_5_ particles with ultrafast and stable Li storage performance.^[^ ^14^, ^15^ ^]^ By far, the majority of studies focus on the modification of closely packed structures, such as orthorhombic phase, high temperature phase and ReO_3_‐type Wadsley–Roth phases, constructed with corner‐shared or edge‐shared octahedrons and tetrahedrons.^[^ ^16^, ^17^ ^]^ Compared with graphite, the relatively dense and rigid structure of Nb_2_O_5_ is less conducive to the rapid Li migration inside the layered structure because of the higher steric hinderance and higher migration barrier. Enlarging the interlayer spacing is beneficial to the charge storage kinetics in intercalation‐type materials.^[^ ^18^, ^19^ ^]^ The electrochemical kinetics of Nb_2_O_5_ based anode still remains to be optimized in such aspects as the homogenization of electron transport and construction of stable Li^+^ channels.

In this paper, we propose a comprehensive strategy of triple conductive wiring through electron doping, chelation coating and electrochemical conversion inside T–Nb_2_O_5_ to achieve a fast‐charging and durable anode for LIBs. This T–Nb_2_O_5_–based anode is firstly doped by Co or Mn heteroatoms to induce the creation of oxygen vacancies to improve its electron conductivity. Afterward, a polydopamine (PDA) layer chelates with and etches the T–Nb_2_O_5_ surface, and it is then pyrolyzed into the exterior carbon coating to further increase the electron conductivity of doped T–Nb_2_O_5_. The PDA etching also triggers the evolution of irregular spheres of T–Nb_2_O_5_ into the well‐defined porous spheres consisting of dendrite‐like primary particles. This fluffy morphology can promote the electrolyte infiltration and sufficient Li‐ion intercalation. The excessive injection of Li‐ions into the lattices of this anode by discharging to a lower voltage allows the in‐situ wiring of conductive NbO domains (with a reported electron conductivity of 4.8 × 10^4^ S cm^−1^ at 300 K) inside the electrochemically cycled T–Nb_2_O_5_, as a result of the phase segregation between NbO and Li‐stuffed Li*_x_* Nb_2_O_5_.^[^ ^20^ ^]^ This new discovery and successful detection of NbO domains explain the much better capacity retention than the case without prior deliberate over‐discharge. The above triple wiring design reinforces the global supply of electron and Li‐ion flows, endowing the modified T–Nb_2_O_5_ anode with ultralong cycling life and high‐rate performance, as well as the potential of high mass loading and high areal capacity. The Li storage mechanism related to the formation and evolution of NbO wiring network is discussed based on in situ structure characterization and electrochemical kinetics analysis in detail.

## 2 Results and Discussion

**Figure 1:**
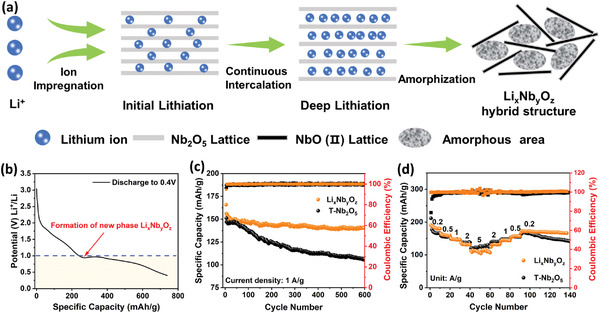
a) Schematic illustration of the formation of Li*_x_* Nb*_y_* O*_z_* hybrid structure. b) The 1st discharge curve of T–Nb_2_O_5_ in a voltage range of 0.4–3 V at 100 mA g^−1^. c) Long‐term cycling performance of pristine T–Nb_2_O_5_ and Li*_x_* Nb*_y_* O*_z_* as anodes at 1 A g^−1^ and d) their corresponding rate performance at different current densities from 0.2 to 10 A g^−1^.

The electrochemical window of T–Nb_2_O_5_ is usually limited to 1–3 V when it is adopted as an anode material in LIB. With the ongoing Li intercalation in the discharge process, the Nb_2_O_5_ particles can become electronically conductive. Here we deliberately broaden the electrochemical window to 0.4–3 V to induce the formation of electronic conduction network inside the micrometer‐scale T–Nb_2_O_5_ particles. The lower cut‐off potential (<0.4 V) is not desirable in view of the risk of Li metal deposition especially under high current density. **Figure** **1**a depicts the formation of NbO electronic conduction domains through electrochemical ion impregnation. The deep lithiation is prone to cause the phase segregation from Li stuffed Li*_x_* Nb_2_O_5_ to the amorphized hybrid of NbO, Li_2_O, and Li*_x_* Nb*_y_* O*_z_*.^[^ ^21^ ^]^ In the voltage range of 1–3 V, the reversible Li storage in T–Nb_2_O_5_ mainly proceeds through a solid solution reaction behavior (Figure 1b). With the progression of Li insertion, the interlayer spacing continues to increase. However, once the voltage reaches 1 V, the newly lithiated phase Li*_x_* Nb*_y_* O*_z_* is formed and the irreversible amorphization is expected to occur. This mechanism will be discussed in detail later. In order to check the influence of Li*_x_* Nb*_y_* O*_z_* on electrochemical cycling, we conducted the initial activation cycling process within 0.4–3 V for 5 cycles to obtain a stable Li*_x_* Nb*_y_* O*_z_* phase. This phase is subsequently cycled at 1A g^−1^ within 1–3 V, and it displays an obviously superior stability over T–Nb_2_O_5_ in Figure 1c. The average capacity decay rate for Li*_x_* Nb*_y_* O*_z_* is as small as 0.017% within 600 cycles, whereas it is 0.051% for T–Nb_2_O_5_ after the same number of cycles. Li*_x_* Nb*_y_* O*_z_* displays the comparable rate performance with that of T–Nb_2_O_5_ in Figure 1d, delivering the reversible capacities of 180, 159, 147, 132, and 112 mAh g^–1^ at 0.2, 0.5, 1, 2, and 5 A g^–1^, respectively. When the current density skips back to 0.2 A g^–1^, the Li*_x_* Nb*_y_* O*_z_* dominant electrode still displays a high capacity of 170 mAh g^–1^ and better cycling stability than T–Nb_2_O_5_. Comparing the charge/discharge curves in the scope of 1–3 V in Figure S1a (Supporting Information), an obvious platform can be observed in Figure 1b below 1 V. The large irreversible capacity below 1 V is mainly ascribed to the Li depletion on carbon black (Figure S1b, Supporting Information). The formation of Li*_x_* Nb*_y_* O*_z_* is also responsible for the partial irreversible capacity. The superiority in electrochemical performance presumably originates from the excellent electrical conductivity of NbO wires and Li*_x_* Nb*_y_* O*_z_* domains, which would be further disclosed below.

**Figure 2:**
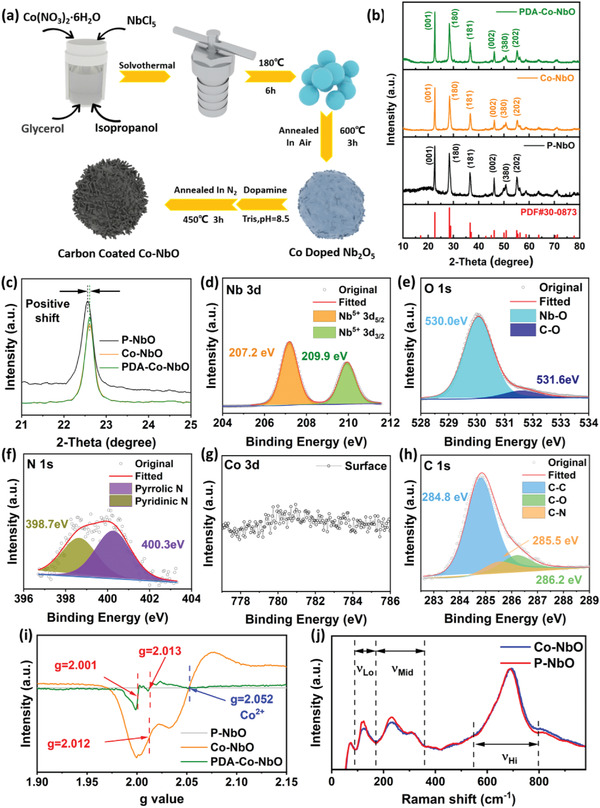
a) Synthetic procedure of PDA–Co–NbO and Co–NbO. b) XRD patterns of P–NbO, Co–NbO, and PDA–Co–NbO, and c) the corresponding magnified XRD patterns in 21°–25°. XPS spectra of d) Nb 3d, e) O 1s, f) N 1s, g) Co 3d, and h) C 1s for PDA–Co–NbO surface. i) EPR curves for P–NbO, Co–NbO, and PDA–Co–NbO. j) Raman spectra for P–NbO and Co–NbO.

To further improve the capacity performance, less than 10 mol % Co or Mn is doped into T–Nb_2_O_5_. **Figure** **2**a depicts a novel bottom‐up synthetic procedure of Co‐doped T–Nb_2_O_5_ (denoted as Co–NbO) and PDA‐derived carbon coated Co–NbO (denoted as PDA–Co–NbO). During the solvothermal reaction in isopropanol, the hydroxyl groups on glycerol enable its complexion with transition metal ions (here including Co^2+^ and Nb^5+^) to form organic–inorganic complex microsphere particles, which ensure the uniform distribution of Co.^[^ ^22^ ^]^ With the subsequent detachment of organic component, the porous structure of Co–NbO is naturally generated. To further fabricate PDA–Co–NbO, we performed the in situ polymerization of dopamine hydrochloride on the Co–NbO surface. Then the PDA conformal coating is carbonized to construct the conformal carbon layer on PDA–Co–NbO with better defined porous structure due to the etching effect of PDA. In Figure 2b, the XRD patterns of Co–NbO and PDA–Co–NbO agree well with the standard diffraction peaks of orthorhombic Nb_2_O_5_ (JCPDS No. 30‐0873, space group: *Pbam*). The pristine T–Nb_2_O_5_ (denoted as P–NbO) is also correspondingly synthesized without the addition of Co precursor as a reference. The most intense peaks at 22.6°, 28.3°, 28.9°, and 36.5° are indexed to (001), (180), (200), and (181) planes, respectively, manifesting the sufficient crystallinity for all three samples.^[^ ^15^ ^]^ Note that no extra peaks of undesirable impurities (e.g., CoO*_x_*) are found for Co–NbO and PDA–Co–NbO, indicating the successful lattice doping of Co into T–Nb_2_O_5_. The bulk doping effect is also disclosed by the positive shifting of XRD peaks in view of the smaller ion radius of Co^2+^ (0.72 Å) or Co^3+^ (0.53 Å) than that (0.78 Å) of Nb^5+^ (Figure 2c). The doping of Co^2+^ with the ion radius comparable to that of Nb^5+^ is beneficial for the preservation of orthorhombic phase. T–Nb_2_O_5_ embraces the layered structure composed of interconnected octahedra (NbO_6_) and pentagonal bipyramids (NbO_7_) through shared corners or edges, which is also called as self‐supporting “room and pillar” structure as shown in Figure S2a (Supporting Information).^[^ ^6^ ^]^ It is deduced that the doped cobalt atoms likely occupy the niobium sites in NbO_6_ and NbO_7_ polyhedra, without the serious change of overall structure framework (Figure S2b,c, Supporting Information). The alternating Nb‐O polyhedron layers along *c* axis are bridged by Nb–O–Nb bonds, and the theoretical interlayer distance of T–Nb_2_O_5_ is 3.93 Å.^[^ ^15^ ^]^ On account of the second‐order Jahn–Teller effect originated from Nb^5+^ (4d^0^), the Nb–O polyhedra in layer are highly distorted, and it complicates the atomic arrangement and cell structure. The partial substitution of cobalt on niobium sites and reduced valence of Co^2+^ (3d^7^) mitigate the cation–cation repulsion and influence the electronic states of Nb^5+^ (4d^0^). Therefore the existence of lower‐valence Co dopant is expected to cushion the Jahn–Teller effect on the distortion of Nb–O polyhedra, and to stabilize Li‐ion migration channels and contribute to the lattice diffusion kinetics.^[^ ^23^, ^24^, ^25^ ^]^

X‐ray photoelectron spectroscopy (XPS) of PDA–Co–NbO was employed to analyze the surface chemical composition. The XPS spectrum of Nb 3d in Figure 2d shows the typical spin–orbit doublet of Nb^5+^, centered at 207.2 eV (Nb^5+^ 3d_5/2_) and 209.9 eV (Nb^5+^ 3d_3/2_), with a spin–orbit splitting energy of 2.7 eV and an approximate peak area ratio of 3:2.^[^ ^26^ ^]^ The O 1s XPS spectrum in Figure 2e can be divided into two peaks centered at 530.0 and 531.6 eV, assigned to Nb–O and C–O, respectively.^[^ ^27^ ^]^ The presence of the smaller shoulder peak is attributed to the residual oxygen during the carbonization of polydopamine or the potential bonding between C coating and lattice O in T–Nb_2_O_5_. The XPS spectrum of N 1s in Figure 2f consist of typical peaks indexed to two different kinds of C–N bonds (at 400.3 eV for pyrrolic N and at 398.7 eV for pyridinic N).^[^ ^28^ ^]^ The doping of trace amount of nitrogen and oxygen in PDA derivative carbon layer is beneficial to improve the lithiophilicity of PDA–Co–NbO surface and promote the uptake and adsorption of electrolyte.^[^ ^29^ ^]^ It is possible that the high adsorption energy of Li on N atoms enables the facile detachment of solvation shell of Li^+^. No obvious peak is observed in the XPS spectrum of Co 3d in Figure 2g, further confirming the successful bulk doping (or burying) of Co rather than surface decoration. The conformal coating of derivative carbon is responsible for the concealing of cobalt signal. The C 1s spectrum in Figure 2h further indicates the dual doping of O and N in C coating layer during PDA pyrolysis from the observation of C–O and C–N peaks at 286.2 and 285.5 eV, respectively.^[^ ^15^, ^28^ ^]^

Electron paramagnetic resonance (EPR) was employed to prove the existence of cobalt, and the magnetic signal of EPR originates from the unpaired electrons. In Figure 2i, the well‐defined peaks can be observed for Co–NbO with two *g* values of 2.012 and 2.052.^[^ ^30^, ^31^ ^]^ Co doping enables the formation of electron‐hole pairs in T–Nb_2_O_5_. The formation of oxygen vacancies resulting from cobalt doping can be described in the following formula

(1) $${\mathrm{Nb}}_{2}O_{5}\overset{\mathrm{CoO}\;\left(+2\right)\;}{\to }2O_{o}+3V_{O}^{\cdot \cdot }+2C{o^{\prime \prime \prime }}_{\mathrm{Nb}}$$

or

(2) $${\mathrm{Nb}}_{2}O_{5}\overset{{\mathrm{Co}}_{2}O_{3}\;\left(+3\right)\;}{\to }3O_{o}+2V_{O}^{\cdot \cdot }+2C{o^{\prime \prime }}_{\mathrm{Nb}}$$

where O_o_ represents the lattice oxygen, $V_{O}^{\cdot \cdot }$ represents the oxygen vacancy with two units of positive charge, Co′′′_Nb_ and Co′′_Nb_ denote the substitution of Co atoms in Nb lattice sites with three and two units of negative charge respectively. The superoxide radicals ($O_{2}^{•-}$) can be formed if the free electrons in electron–hole pairs are captured by oxygen atoms as described in the following formula

(3) $$2O_{o}+C{o^{\prime \prime \prime }}_{\mathrm{Nb}}\;\to O_{2}^{\cdot -}+C{o^{\prime \prime }}_{\mathrm{Nb}}$$

The peak at *g* = 2.012 should be associated with the existence of superoxide radicals ($O_{2}^{•-}$).^[^ ^32^ ^]^ And the peak at *g* = 2.052 should stem from the unpaired electrons on cobalt, e.g. three unpaired electrons in Co^2+^ and four unpaired electrons in Co^3+^.^[^ ^33^ ^]^ In this case the magnetic signal coming from cobalt likely influences the detection of superoxide radicals, leading to a subtle positive shift of *g* value. In the EPR curve of PDA–Co–NbO, there are two obvious peaks centered at *g* = 2.001 and *g* = 2.013. The elimination of peaks corresponding to cobalt possibly originates from the electromagnetic‐interference shielding of surface carbon layer in PDA–Co–NbO.^[^ ^34^ ^]^ Both the signals at *g* = 2.001 and 2.013 should be associated with the stabilization of superoxide radicals but with different coordination environments, but they are much weaker than that at *g* = 2.012 for Co–NbO. The second annealing for PDA pyrolysis in inert atmosphere is responsible for the healing of excess $O_{2}^{•-}$ and therefore the attenuation of EPR signals. Note that the peak at *g* = 2.001 is probably assigned to the stabilization of oxygen radicals anchored at the interface with O/N doped carbon coating.^[^ ^35^ ^]^ Raman spectra were used to explore the vibration situations of metal–oxygen bonds in Co–NbO as shown in Figure 2j. Both the spectra of P–NbO and Co–NbO are nearly identical. The three peaks in 100–400 cm^–1^ correspond to typical bending modes of Nb–O–Nb linkages.^[^ ^36^ ^]^ After Co doping, the intensities of the two peaks at 120 and 230 cm^–1^ are obviously weakened, in view of the potential formation of Co–O–Nb linkages and the resultant disordering of bending in T–Nb_2_O_5_. The strong and broad peak at 690 cm^–1^ corresponds to the symmetric stretching mode of NbO_6_ and NbO_7_.^[^ ^36^ ^]^ Note that no additional peaks assigned to the stretching of Co–O bonds is observed in the range of 300–800 cm^–1^,^[^ ^37^ ^]^ further verifying the suppression of CoO*_x_* phase precipitation and firm substitution of cobalt atoms on niobium lattice sites in Co–NbO, which benefits from the excellent dispersion effect of Co element in organic–inorganic complex precursor. This EPR result indicates that the Co doping enables the stimulation of free electrons, enabling the improvement of bulk electron conductivity in PDA–Co–NbO and Co–NbO.

**Figure 3:**
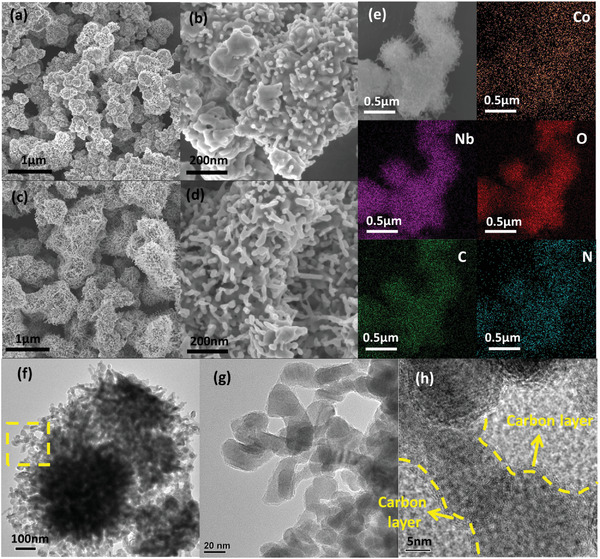
Overview and magnified SEM images of a,b) Co–NbO and c,d) PDA–Co–NbO. e) Corresponding EDS mapping analysis of Co, Nb, O, C, and N elements for PDA–Co–NbO particles. f) TEM image of PDA–Co–NbO and g) magnified image of the selected region in yellow square. h) HRTEM image of carbon layers on the surface of PDA–Co–NbO particles.

The field emission scanning electron microscopy (SEM) images of Co–NbO (**Figure** **3**a,b) display the euhedral moss‐like primary particles with a dimension around 18 nm on average, which are self‐assembled into the secondary particles with the size ranging from 500 nm to 1 µm. The extra pyrolyzed PDA coating enables the etching effect of PDA–Co–NbO grains from the SEM imaging in Figure 3c,d. Although the size of secondary particles of PDA–Co–NbO (0.5–1 µm) is comparable to that of Co–NbO, the surface and bulk porosity becomes much more remarkable for PDA–Co–NbO with the appearance of impressive coral‐like morphology with obvious dendritic protrusions. The fluffy PDA–Co–NbO as anode material is beneficial for the electrolyte penetration and uptake, while the PDA derivative carbon layer on each dendritic protrusion can homogenize and interconnect the electron paths. The chelation of niobium cation with PDA is responsible for the etching of niobium oxide particles and firm construction of carbon conformal layer,^[^ ^38^ ^]^ which effectively retards the fusion of adjacent particles and optimize the porosity of electrode network. The elemental mapping by energy dispersive X‐ray spectroscopy (EDS, Figure 3e) displays the homogeneous distribution of Nb, O, Co, C, and N elements in fluffy PDA–Co–NbO particles. The conformal effect of N‐doped carbon coating can be confirmed from the similar mapping profiles of C, N, and Nb. The cobalt signal is relatively weaker due to its lower molar content in bulk T–Nb_2_O_5_. The transmission electron microscopy (TEM) imaging was used to further explore the detailed structural and morphology information of PDA–Co–NbO in Figure 3f–h and Figure S3 (Supporting Information). One can find that the primary particles are interconnected with each other through the intricate intergrowth of dendritic protrusions. These protrusions are actually composed of the smaller margin‐eutectic nanoscale particles with the dimension around 20 nm. The high resolution TEM (HRTEM) imaging (Figure S3b, Supporting Information) displays the well‐defined lattice fringes in the primary particles with the interlayer spacings of 3.927 and 3.139 Å, which are indexed to typical (001) and (180) planes of doped T–Nb_2_O_5_, respectively. The uniform carbon layer with a thickness of ≈2 nm can be discerned to conformally decorate on the surface of T–Nb_2_O_5_ nanoparticles from the HRTEM images as well (Figure 3h and Figure S3c, Supporting Information). The TEM result well discloses the sufficient porosity shaped by numerous dendritic protrusions of PDA–Co–NbO based on the self‐etching mechanism of Co–NbO template via the chelation of Nb^5+^ nodes by the catechol moieties in PDA layer.^[^ ^38^ ^]^

**Figure 4:**
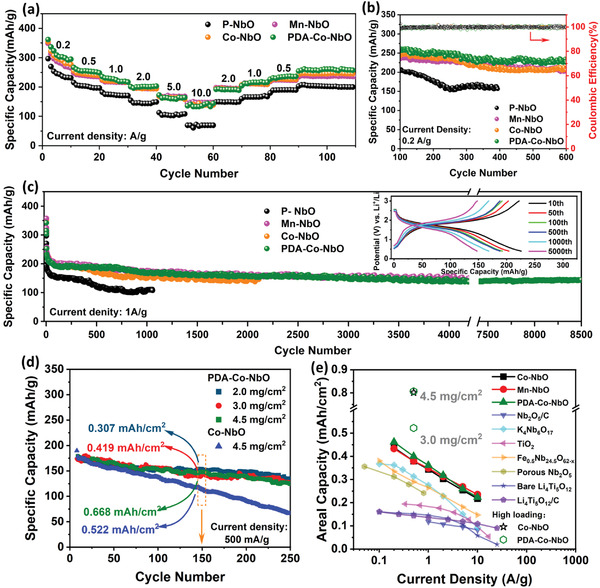
a) Rate performance comparison of Li/P–NbO, Li/Co–NbO, and Li/PDA–Co–NbO cells at different current densities ranging from 0.2 to 10 A g^−1^. b) Cycling performance comparison of corresponding cells at 0.2 A g^−1^ after rate performance testing. c) Long‐term cycling performance comparison of Li/P–NbO, Li/Co–NbO, and Li/PDA–Co–NbO cells in a voltage range of 0.4–3.0 V at 1 A g^−1^. Inset: corresponding charge/discharge curves of PDA–Co–NbO at the 10th, 50th, 100th, 500th, 1000th, and 5000th cycles. d) Cycling performance of Co–NbO and PDA–Co–NbO under different mass loadings. e) Areal capacity comparison of our PDA–Co–NbO, Co–NbO with other typical anode materials, including T–Nb_2_O_5_, TiO_2_, Li_4_Ti_5_O_12_, and K_4_Nb_6_O_17_, at different current densities and different mass loadings.

The electrochemical activation process in P–NbO mentioned in Figure 1 is also applicable to Co–NbO, PDA–Co–NbO as well as other heteroatom doped T–Nb_2_O_5_ (e.g., Mn–NbO). The electrochemical window of T–Nb_2_O_5_ was usually confined to 1–3 V in most literatures, which corresponds to two Li^+^ insertion in one Nb_2_O_5_ unit.^[^ ^39^ ^]^ Here the electrochemical window is extended to 0.4–3.0 V to improve the specific capacity. The corresponding galvanostatic charge/discharge tests of Li/P–NbO, Li/Co–NbO, Li/PDA–Co–NbO and Li/Mn–NbO cells were performed to testify the extra contributions of Co/Mn dopant and conductive carbon layer on cycling performance. **Figure** **4**a displays the rate performance of P–NbO, Co–NbO, PDA–Co–NbO and Mn–NbO anodes at the current densities from 0.2 to 10 A g^–1^. PDA–Co–NbO displays the higher specific capacities at all the current densities (≈292.2, 255.1, 228.4, 201.1, 164.4, and 144.1 mAh g^–1^ at 0.2, 0.5, 1.0, 2.0, 5.0 and 10.0 A g^–1^, respectively, Figure S4, Supporting Information), whereas the corresponding capacity values for P–NbO are 231.2, 201.8, 171.6, 144.2, 104.1, and 69.4 mAh g^–1^. When the current density is restored to 0.2 A g^–1^ (Figure 4b), the PDA–Co–NbO anode delivers not only the better cycling stability but also the higher specific capacity (255 mAh g^–1^) than that of Co–NbO (246 mAh g^−1^). During the long‐term cycling for at least 600 cycles, the coulombic efficiency (CE) plots are highly stabilized at 100%. It is evident that the extra wiring of conformal carbon layer endows PDA–Co–NbO with better cycling durability, which is also supported by the superior long‐term cycling performance at a higher current density of 1 A g^−1^ in Figure 4c (with a precycling process at 0.2 A g^−1^ for three cycles). It needs to be emphasized that the Li/PDA–Co–NbO cell can preserve a specific capacity as high as 143 mAh g^−1^ after cycling for extra‐long 8500 cycles. Within the 1st–2000th cycles, Li/PDA–Co–NbO cell exhibits the obviously better capacity retention (69%) than Li/Co–NbO cells (57%). The corresponding charge/discharge curves of Li/PDA–Co–NbO cell display the high symmetry and excellent reversibility even after cycling for 5000 cycles (inset of Figure 4c). Our PDA–Co–NbO anode characterized by triple wiring of chelation coating, electron doping and electrochemical conversion below 1 V enables the impressive reversibility and it is expected to become a promising candidate for durable anode materials. When we stepwisely increase the areal loading from 2 to 4.5 mg cm^−2^, PDA–Co–NbO also enables a much better capacity retention (than Co–NbO) with areal capacities of 0.307, 0.419 and 0.668 mAh cm^−2^ at the areal loadings of 2.0, 3.0, and 4.5 mg cm^−2^, respectively at the 150^th^ cycle and 500 mA g^−1^ (Figure 4d). When the mass loading increases up to 4.5 mg cm^−2^, PDA–Co–NbO still displays a capacity decaying rate as small as 0.12%, much lower than that of Co–NbO (0.26%) within 250 cycles. The wiring and infiltration effects of firm conformal carbon layer at the surface of each primary particle of PDA–Co–NbO are responsible for the preservation of high areal capacity even under the high loading and long cycling conditions. The areal capacity advantage of PDA–Co–NbO at different current densities is displayed when comparing with other representative intercalation anodes (such as TiO_2_, Li_4_Ti_5_O_12_, T–Nb_2_O_5_, K_4_Nb_6_O_17_ and so on) in Figure 4e, which also highlights the excellent rate performance of PDA–Co–NbO.^[^ ^40^, ^41^, ^42^, ^43^, ^44^, ^45^ ^]^ This areal capacity value of 0.807 mAh cm^−2^ for PDA–Co–NbO are more than two times of those for other typical anode materials at the same current density of 500 mA g^−1^.

**Figure 5:**
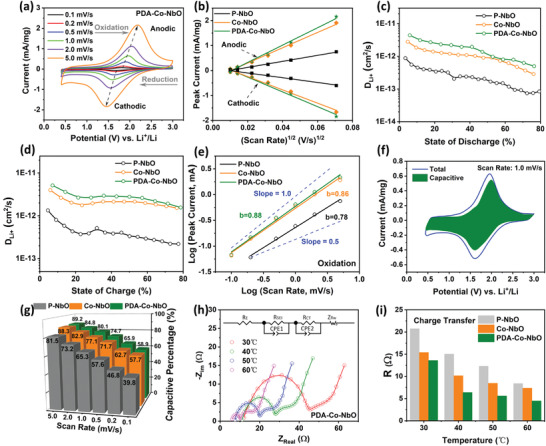
a) CV curves of PDA–Co–NbO at incremental scan rates from 0.1 to 5 mV s^−1^. b) Linear fitting plots of peak current versus the square root of scan rate for P–NbO, Co–NbO and PDA–Co–NbO during both cathodic and anodic processes. Li‐ion diffusion coefficients calculated from GITT for P–NbO, Co–NbO, and PDA–Co–NbO as a function of c) state of discharge and d) state of charge. e) Linear fitting plots of power law dependence based on log *i*(*V*) = *b*log *v* + log *a*, where *v* is the scan rate and *i*(*V*) refers to the corresponding peak current at a given voltage position (*V*). f) CV curve of PDA–Co–NbO at 1.0 mV s^−1^ with the corresponding pseudocapacitive current contribution outlined into green region. g) 3D column graphs of pseudocapacitive contribution fraction (%) in charge storage at different scan rates from 0.1 to 5.0 mV s^−1^ for P–NbO, Co–NbO, and PDA–Co–NbO. h) Nyquist plots and their equivalent circuit fitting of PDA–Co–NbO at different temperatures from 30 to 60 ℃, inset: corresponding equivalent circuit. i) Column graphs of charge transfer resistance for P–NbO, Co–NbO, and PDA–Co–NbO at different test temperatures.

As revealed in **Figure** **5**a and Figure S5 (Supporting Information), the typical cyclic voltammetry (CV) curves of P–NbO, Co–NbO and PDA–Co–NbO display one pair of regular redox peaks with the scan rates from 0.1 to 5 mV s^−1^, corresponding to the charge/discharge platforms in Figure 4. With the stepwise increase of scan rate, the potential gap between anodic and cathodic peaks in CV curves also correspondingly becomes larger. Li/PDA–Co–NbO cells display lowest potential gap at all rates except 5.0 mV s^−1^, while Li/Co–NbO cells embrace highest potential gap at all rates except 0.1 mV s^−1^. Figure 5b indicates the linear fitting relationship between the peak current and the square root of scan rate (V s^−1^), and the steeper slope reflects the higher diffusion coefficient according to Formula S1 (Supporting Information). Obviously, the peak current responses and corresponding slopes for PDA–Co–NbO and Co–NbO highly exceed those for P–NbO, benefiting from the superior electrochemical kinetics for the former two. The higher diffusion coefficient (proportional to the slope of fitting line) is attributed to the synergistic contributions of conductive carbon layer network, NbO domains and cobalt dopants. The lithium diffusion coefficient can be quantitatively estimated by the galvanostatic intermittent titration technique (GITT), based on the following formula

(4) $$D=\frac{4}{\pi }{\left(\frac{m_{B}V_{m}}{M_{B}S}\right)}^{2}{\left(\frac{\Delta E_{S}}{\tau \left(dE_{\tau }/d\sqrt{\tau }\right)}\right)}^{2}\left(\tau \ll L^{2}/D\right)$$

where *D* is the Li diffusion coefficient, *S* and *L* refer to the thickness and surface area of electrode material, respectively, *m*_B_, *V*_m_, and *M*_B_ are denoted as the mass loading, theoretical molar volume (50.7 cm^3^ mol^−1^) and theoretical molecular weight (265.81 g mol^−1^) of T–Nb_2_O_5_, respectively.^[^ ^46^ ^]^ As visualized in the GITT curves (Figure S6a, Supporting Information) and enlarged plots (Figure S6b, Supporting Information), Δ*E*_S_ is the gap of potentials between two adjacent open‐circuit states after relaxation for 6 h, and *τ* refers to the intermittent charge/discharge time (3600 s). The *E*_*τ*_ value is equal to the transient potential during the pulsing step and it displays a linear relationship (*R* ^2^ = 0.998) with the square root of pulsing time (Figure S6c, Supporting Information). Therefore, the above‐mentioned formula can be simplified into the following equation

(5) $$D=\frac{4}{\pi \tau }{\left(\frac{m_{B}V_{m}}{M_{B}S}\right)}^{2}{\left(\frac{\Delta E_{S}}{\Delta E_{\tau }}\right)}^{2}\left(\tau \ll L^{2}/D\right)$$

The calculated Li diffusion coefficients (e.g., ranging from 1.56 × 10^–12^ to 5.13 × 10^–12^ cm^2^ s^−1^ during charge and from 4.97 × 10^–13^ to 4.40 × 10^–12^ cm^2^ s^−1^ during discharge for PDA–Co–NbO) are roughly two order of magnitude higher than those of fast‐charging type Li_4_Ti_5_O_12_ (6.77 × 10^–14^ to 2.8 × 10^–15^ cm^2^ s^−1^) reported in literatures (Figure 5c,d).^[^ ^47^ ^]^ The D values of tailored PDA–Co–NbO and Co–NbO are evidently higher than those of P–NbO at all the tested states of charge and discharge (denoted as SOC and SOD, respectively). When SOC and SOD are lower than 80%, the D values for PDA–Co–NbO are higher than for Co–NbO and they display an overall downward tendency with the increase of SOC and SOD. These phenomena indicate that both the deep lithiation and delithiation would somewhat degrade the electrochemical kinetics. The Li‐stuffed and Li‐emptied channels likely become less stable or more distorted as observed from the potential conversion into NbO phase and amorphization of lithiated Li*_x_* Nb*_y_* O*_z_* phase, which would be further discussed below.

The intercalation pseudocapacitance should be also responsible for the high‐rate performance of PDA–Co–NbO and Co–NbO, and it was quantitatively analyzed based on the collected CV curves at different scan rates. The dominant electrochemical step can be primarily judged through the assumed power‐law relationship of peak current versus sweep rate (*i* = *av^b^*).^[^ ^48^ ^]^ When the b value is close to 0.5, the current response is controlled by semi‐infinite linear diffusion, and the surface capacitance dominates the current response when the b value approaches 1. As shown in Figure 5e and Figure S7a (Supporting Information), the PDA–Co–NbO and Co–NbO electrodes enable the higher b values (0.88 and 0.86 for oxidation process, 0.86 and 0.85 for reduction, respectively) than those for P–NbO (0.78 and 0.77 for oxidation and reduction, respectively). Furthermore, the total current response can be distinctly separated into intercalation current (*k*_1_ *v*) and pseudocapacitive current (*k*_2_ *v* ^1/2^) at a given voltage (*V*) according to the following equation

(6) $$i\left(V\right)=k_{1}v+k_{2}v^{1/2}$$

namely

(7) $$i\left(V\right)/v^{1/2}=k_{1}v^{1/2}+k_{2}$$

Therefore, the parameters *k*_1_ and *k*_2_ can be obtained through the linear relationship of *i*(*V*)/*v* ^1/2^ versus *v* ^1/2^.^[^ ^49^, ^50^ ^]^ The calculated pseudocapacitive current plots (vs potential) within the total CV curve can be integrated into the green area as shown in Figure 5f (for PDA–Co–NbO at 1.0 mV s^−1^), and the corresponding areas for P–NbO and Co–NbO are outlined in Figure S7b,c (Supporting Information). As columned in Figure 5g, the pseudocapacitive contributions of charge storage in PDA–Co–NbO and Co–NbO are obviously greater than that in P–NbO at all the sweep rates (e.g., 80.1% and 77.1% vs 65.3% at 1.0 mV s^−1^). This enhancement is in accordance with the better rate performance for the formers, and is ascribed to the coral‐like porous electrode structure (with higher specific area surface), defect and coating reinforced charge transport pathways.^[^ ^51^, ^52^ ^]^

Electrochemical impedance spectroscopy (EIS) was employed to further explore the evolution of electrochemical kinetics after the introduction of cobalt and carbon layer. Before the EIS measurement, the Li/PDA–Co–NbO, Li/Co–NbO and Li/P–NbO cells were cycled for 5 cycles at 100 mA g^−1^ in advance to stabilize the solid electrolyte interphase (SEI). The obtained Nyquist plots with frequencies from 1 MHz to 0.01 Hz and at stepwise increasing temperatures (30 – 60 ℃) are depicted in Figure 5h, Figure S8a,b (Supporting Information). The Nyquist plots are fitted with the equivalent circuit displayed in the inset of Figure 5h according to our previous reports.^[^ ^53^ ^]^ The typical well‐defined semicircle at high frequencies is assigned to the resistance of solid electrolyte interface (denoted as *R*_SEI_), the incomplete semicircle at medium‐frequency range is attributable to the change transfer resistance (denoted as *R*_CT_), and the steep tail (nearly straight line) at low frequency zone is simulated with a finite‐length Warburg impedance component (*Z*_flw_), which presents Li^+^ diffusion and accumulation at the electrolyte/electrode interface.^[^ ^54^ ^]^ The specific frequency ranges of above‐mentioned three regions can be found in Figure S8c (Supporting Information). The intercept on real axis results from the bulk electrolyte resistance (*R*_E_), and there is almost no difference of *R*_E_ values (usually below 8 Ω) among all the three cells. The Li/PDA–Co–NbO cell enables the smallest resistance contributions from charge transfer and SEI, and therefore the smallest total interface resistance R_Total_ (Figure 5i and Figure S8d,e, Supporting Information) at various temperatures. The remarkable decrease of interface resistance is associated with the better interface wettability promoted by the doped surface and carbon coating. The nanostructuring and etching effects on PDA–Co–NbO and Co–NbO also enable the shrinkage of interface resistance in view of the more sufficient contact between active grains and electrolyte, which does not passivate the grain surface due to the stable voltage window above 0.4 V. Note that when enhancing the testing temperature to 60 °C, the *R*_SEI_ value for P–NbO becomes substantially smaller than that for Co–NbO, indicating that the less penetrative SEI in P–NbO is relatively vulnerable and is prone to be thermally fractured. The SEI networks in PDA–Co–NbO and Co–NbO are more robust in a wider temperature range.

**Figure 6:**
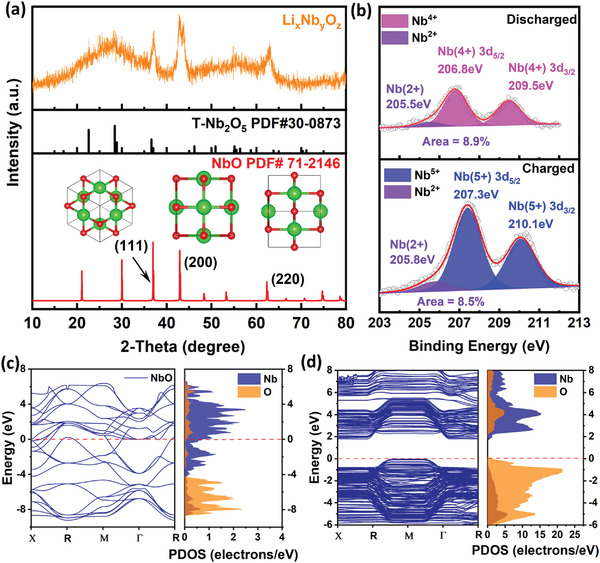
a) XRD pattern of lithiated phase Li*_x_* Nb*_y_* O*_z_* after 5 cycles. Inset: crystal structures of NbO along [111], [200] and [220] directions. b) XPS spectra of Nb 3d for fully discharged and charged Li*_x_* Nb*_y_* O*_z_* electrodes. Calculated energy band structure (left) and partial density of states (right) of c) NbO and d) T–Nb_2_O_5_.

Transition metal dopants (e.g., Co, Mn) and nitrogen doped carbon layer can synergistically endow the T–Nb_2_O_5_ particles with better electrochemical activity. To clarify the structural evolution, a series of characterizations on the cycled electrodes were adopted as shown in **Figure** **6**. The XRD pattern of lithiated product can be assigned to cubic niobium monoxide (NbO) (JCPDS No. 71‐2146, space group: *Pm3m*).^[^ ^55^ ^]^ What is more, a broad peak can be observed ranging from 20 to 35°, and it can be ascribed to the amorphization of Li*_x_* Nb*_y_* O*_z_* (Figure 6a).^[^ ^56^ ^]^ The XPS spectra (Figure 6b) of cycled Co–NbO at the fully charged and discharged states also indicate the formation of NbO (from the Nb 3d peak at 205.5 eV) in the discharged process.^[^ ^21^ ^]^ At the fully charged state, the NbO peak signal still exists with the similar peak area ratio (8.5%), indicating the electrochemical irreversibility of NbO conversion product, which is expected to serve as electronically conductive wires to promote the cycling stability of Li*_x_* Nb*_y_* O*_z_*. Note the fraction of this nonactive phase is not high and therefore the capacity performance would not be compromised. This result also implies that the primary Nb_2_O_5_ would not undergo the deep conversion reaction and the appearance of NbO is likely caused by the shallow phase segregation of Li‐stuffed Li*_x_* Nb_2_O_5_ or Li*_x_* Nb*_y_* O*_z_* into NbO and Li_2_O.^[^ ^21^ ^]^ The local phase precipitation is sufficient to alleviate the lattice stress of Li*_x_* Nb*_y_* O*_z_* and retard its further decomposition. The electrochemical capacity release should stem from the formation of dominant Nb^4+^ peaks (at 206.8 eV for 3d_5/2_ and 209.5 eV for 3d_3/2_) at the discharge state.^[^ ^57^ ^]^ The Nb^4+^ species can be reversibly converted into the Nb^5+^ species (with dominant peaks at 207.3 eV for 3d_5/2_ and 210.1 eV for 3d_3/2_) after recharging.^[^ ^57^, ^58^ ^]^ Therefore, the lithium storage mainly depends on the reversible conversion between Nb^5+^ and Nb^4+^, and the NbO domains mainly assist the migration of electron flow. The partial density of states (PDOS) and corresponding band structures of NbO and T–Nb_2_O_5_ were calculated through density function theory (DFT) to unveil the electrical conductivities of NbO and T–Nb_2_O_5_ in Figure 6c,d. The Femi surface is marked with dotted line. No band gap can be observed for NbO, while the calculated bandgap of T–Nb_2_O_5_ is 1.75 eV. The conductive NbO has the overlapped conduction band (CB) and valence band (VB), since both the CB and VB are composed of Nb 3d orbitals. The calculated PDOS of T–Nb_2_O_5_ shows that VB is mainly composed of O 2p and Nb 4p orbitals, while CB is mainly composed of Nb 3d orbitals. This result strongly proves that the cycled electrode of NbO/Li*_x_* Nb*_y_* O*_z_* displays the obviously higher electrical conductivity than T–Nb_2_O_5_. The calculated XRD patterns of NbO and T–Nb_2_O_5_ structures used for PDOS calculation are displayed in Figure S9a (Supporting Information), and they are consistent with the XRD patterns of experimentally obtained NbO and T–Nb_2_O_5_ phases. The in situ Raman spectra (Figure S9b, Supporting Information) are used to reveal the structural evolution during cycling. The peaks at about 230 cm^–1^ are assigned to the typical bending modes of Nb–O–Nb linkages,^[^ ^36^ ^]^ and they do not undergo evident shifting from 1.0 to 0.4 V. This phenomenon further indicates that the large irreversible capacity below 1 V is mainly ascribed to the Li depletion on carbon black rather than the excess reduction of most Nb–O–Nb moieties (Figure S1b, Supporting Information). During the following charge, a gradual positive shifting is observed, and it is ascribed to the shortening of metal–oxygen bonds caused by the oxidation of Nb–O–Nb moieties with the Nb valence state increase from +4 to +5.^[^ ^59^ ^]^ The peaks around 470 cm^−1^, corresponding to the vibrations of electrolyte molecules (ethylene carbonate and dimethyl carbonate),^[^ ^7^ ^]^ do not undergo the position shifting during the whole cycling. The shallow conversion reaction into NbO and irreversible Li consumption by carbon additive below 1 V do not degrade the electrolyte stability.

**Figure 7:**
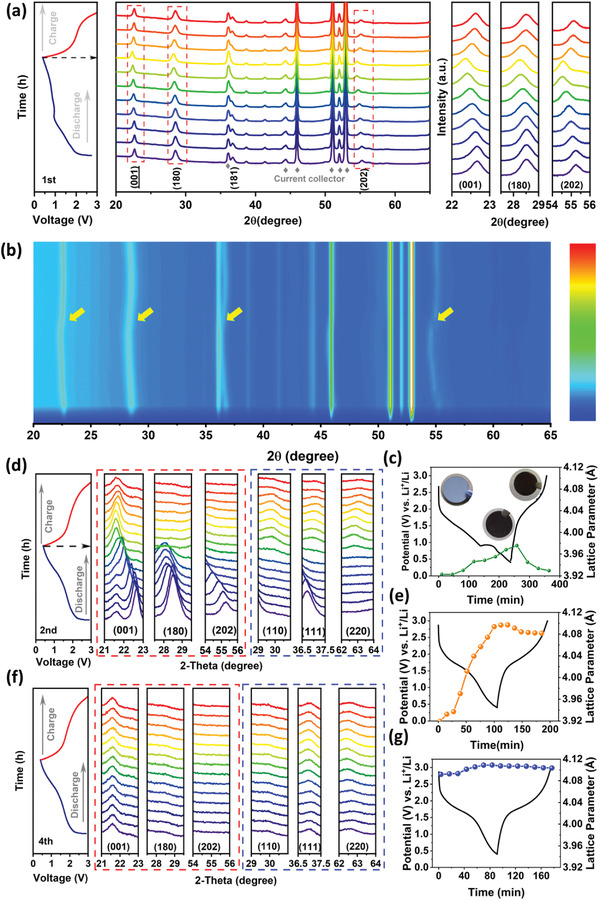
a) In situ XRD characterization and stacked XRD patterns for PDA–Co–NbO anode during the first cycle, and b) the corresponding contour plots and c) the corresponding lattice parameter evolution plots. d) The evolution of selected XRD peaks and e) corresponding lattice parameter plots during the second cycle. (f) The evolution of selected XRD peaks and g) corresponding lattice parameter plots during the fourth cycle. The selected peaks in red square are assigned to T–Nb_2_O_5_ and the peaks in blue square are assigned to NbO.

The in situ XRD measurements were performed on the PDA–Co–NbO electrode within the first four charge/discharge cycles to further reveal the detailed structural evolution and the formation of NbO. **Figure** **7**a records the in situ XRD patterns of PDA–Co–NbO during the first cycle, and the corresponding voltage profiles are displayed on the left. Besides the six peaks assigned to current collector (marked with gray rhombus), the four dominant diffraction peaks at 22.5°, 28.4°, 36.6°, and 55.1° correspond to (001), (180), (181), and (202), respectively, consistent with the standard diffraction peaks of orthorhombic niobium pentoxide. In the first discharge process, the positions of all the (001), (180), (181), and (202) peaks gradually shift to the lower angles with continuous lithium intercalation. This negative shifting is caused by the lattice expansion with the increase of cationic radius from Nb^5+^ (0.78 Å) to Nb^4+^ (0.83 Å). On the other hand, the lattice expansion may also be caused by the lithium occupation on the interstitial sites of NbO_7_/NbO_6_ polyhedra. In the following charge process, the highly reversible positive shifting is observed with lithium extraction. The in‐situ XRD evolution indicates that the dominant electrochemical behavior is the reversible solid‐solution‐like (de)lithiation without the obvious appearance of phase transformation during the first cycle. The excellent reversibility of XRD pattern evolution is also illustrated by the corresponding 2D contour plots in Figure 7b. Note that the lattice parameter on c axis, namely, the interlayer spacing of (001) planes, still undergoes a gradual increase with the progression of lithiation time even after the voltage drops below 1 V. This phenomenon should stem from the slower recording of XRD signals behind the accurate lithiation process. Therefore the potential formation of NbO below 1 V cannot be promptly revealed. Of course, its poor crystallinity is also responsible for the difficulty to detect NbO phase during the first cycle. This lattice parameter fluctuates reversibly between 3.92 and 3.98 Å with the insertion and extraction of Li^+^, agreeing with the solid solution behavior (Figure 7c). Note that the lattice parameter (3.93 Å) for the fully charged electrode is slightly larger than that (3.92 Å) of the primary electrode. The expanded channels would destabilize the “room and pillar” structure during the following cycles as discussed below.

In contrast, in the second discharge process (Figure 7d, red dotted frame), the diffraction peaks of (180) and (202) become gradually broader and finally vanish as they move toward the lower angles. These peaks do not recover during the following charge, confirming the irreversible amorphization of PDA–Co–NbO. The potential lattice Li residual and stoichiometric evolution in the first charged product (T–Nb_2_O_5_‐like phase) are likely responsible for the acceleration of structure disorder, although it is still based on the solid solution reaction behavior. The stoichiometric loss should be triggered by the phase segregation from Li‐stuffed Li_2_Nb_2_O_5_ with the formation of NbO and Li_2_O domains. The (001) peak signal can be preserved with less broadening and it does not vanish during the following charge, but it cannot return to the original position before the beginning of the second cycle. Instead, this (001) peak experiences a marginal shifting during the second charge. It appears that the “room and pillar” constructed layers along c axis are more resistant to the structure collapse or disordering. The corresponding lattice parameter undergoes an increase from 3.92 to 4.10 Å with a larger magnitude during the second discharge compared with the case in the first discharge (Figure 7e). This further expanded interlayer would no longer shrink substantially during the following Li extraction as observed from the slow decrease of lattice parameter merely from 4.10 to 4.08 Å, leading to an unusual zero‐strain‐like charging behavior. This behavior would dominate the following electrochemical cycles (e.g., with marginal evolution of lattice parameter around 4.10 Å in the whole 4^th^ cycle as shown in Figure 7f,g). The zero‐strain lithiation is favorable for the ultralong cycling performance for at least 8500 cycles.^[^ ^49^ ^]^ Note that three new peaks (at 30°, 37°, and 62.3°) appear during the second charging process, respectively corresponding to the (110), (111), and (220) planes of NbO in Figure 7d (blue dotted frame). These peaks are still discernable during the 4^th^ cycle, as the once‐formed NbO domains would not be electrochemically oxidized and they only serve as electronic wires. It is speculated that the intercalation of a large amount of lithium incurs the irreversible lattice expansion of Nb_2_O_5_, leading to the partial structural disorder of T–Nb_2_O_5_ with the formation of more crystallized NbO and less crystallized Li*_x_* Nb*_y_* O*_z_* at the same time, similar to the report of disordered rock salt Li_3_V_2_O_5_ phase as the lithiation product of V_2_O_5_.^[^ ^60^ ^]^

**Figure 8:**
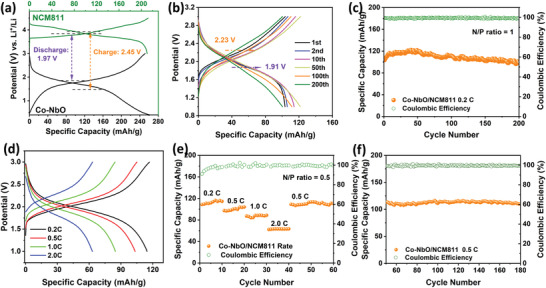
a) Charge/discharge voltage profiles of Co–NbO anode at 200 mA g^−1^ and NCM811 cathode at 56 mA g^−1^. b) Charge/discharge curves of Co–NbO/NCM811 full cell at different cycling stages at 0.2 C (1 C = 280 mA g^−1^). c) Corresponding cycling performance of Co–NbO/NCM811 full cell with a N/P ratio of 1. d) Charge/discharge curves of Co–NbO/NCM811 full cell at different rates from 0.2 to 2.0 C. e) Corresponding rate performance of Co–NbO/NCM811 full cell with a N/P ratio of 0.5. f) Cycling performance of Co–NbO/NCM811cell at 0.5 C after rate performance testing.

Since the PDA–Co–NbO electrode would consume more irreversible Li by the carbon coating network,^[^ ^61^, ^62^ ^]^ we used Co–NbO as anode to verify the practical availability of assembled Co–NbO/LiNi_0.8_Co_0.1_Mn_0.1_O_2_ (NCM811) and Co–NbO/LiFePO_4_ full cells. After the cycling of Co–NbO at 100 mA g^−1^ for 5 cycles in advance (with lithium as counter electrode), the SEI‐stabilized Co–NbO was disassembled and adopted as anode in full cells. The typical charge/discharge curves of Co–NbO anode and NCM811 cathode in **Figure** **8**a indicate that the theoretical output voltages of Co–NbO/NCM811 full cells should be ≈2.45 V and 1.97 V in the charge and discharge processes, respectively (Figure S10a–d, Supporting Information), close to the corresponding practical voltages (2.23 and 1.91 V) obtained from the charge/discharge curves of Co–NbO/NCM811 full cells (Figure 8b). The practical voltage is estimated based on the integrated area of voltage versus capacity in Figure S10e,f (Supporting Information). The cycling performance of Co–NbO/NCM811 full cell is highly stable with high CE of 100% even under a low N/P ratio of 1 (Figure 8c). Its discharge capacity is still as high as 95 mAh g^−1^ after 200 cycles with an average decaying rate as small as 0.09% per cycle at a rate of 0.5 C (1C = 280 mA g^−1^) at room temperature. The Co–NbO/NCM811 full cell delivers the reversible capacities of 116.3, 103.6, 88.7, 63.0 mAh g^−1^ at the rates of 0.2, 0.5, 1.0, and 2.0 C, respectively under a smaller N/P ratio of 0.5 (Figure 8d,e). When the rate returns to 0.5 C, this full cell still exhibits a reversible capacity around 110 mAh g^−1^ after 180 cycles (Figure 8f). The practicability of Co–NbO anode is also verified by the operation of Co–NbO/LiFePO_4_ full cells with well‐defined reaction plateaus, stable cycling and satisfactory rate performance as shown in Figures S11 and S12 (Supporting Information).

## 3 Conclusion

In summary, we propose a triple conductive wiring strategy through electron doping, chelation coating and electrochemical conversion inside the micro‐sized aggregates of T–Nb_2_O_5_ particles to achieve the fast‐charging and durable anodes for LIBs. The penetrative coating of conformal carbon layer and formation of NbO domains in discharged T–Nb_2_O_5_ promote the global enrichment of electronically conductive wires, leading to the much better capacity retention than pristine T–Nb_2_O_5_ and conventionally doped one. This unique triple wiring endows PDA–Co–NbO anode with extraordinary stable cycling performance (143 mAh g^−1^ at 1 A g^−1^ after 8500 cycles) and high‐rate performance (144.1 mAh g^−1^ at 10.0 A g^−1^). The multiple wiring effect also enables a high mass loading of 4.5 mg cm^−2^ and high areal capacity of 0.668 mAh cm^−2^ (even after 150 cycles) for PDA–Co–NbO. The exploration of more conductive wiring models in T–Nb_2_O_5_ is a potential solution to its practical application in high‐rate LIBs.

## Conflict of Interest

The authors declare no conflict of interest.

## Supporting information

Supporting Information

## Data Availability

The data that support the findings of this study are available from the corresponding author upon reasonable request.
