# Supplementary material for: Triple Conductive Wiring by Electron Doping, Chelation Coating and Electrochemical Conversion in Fluffy Nb2O5 Anodes for Fast‐Charging Li‐Ion Batteries
Source: Adv Sci (Weinh). 2022 Jul 7;9(25):2202201. doi: 10.1002/advs.202202201 (PMC9443447; doi:10.1002/advs.202202201)
Supplement: Supplementary file 1 — Supporting Information [file ADVS-9-2202201-s001.pdf]

Supporting Information

Triple conductive wiring by electron doping, chelation coating and electrochemical conversion in fluffy Nb_2O_5 anodes for fast-charging Li-ion batteries

Yongjian Zheng[†], Wujie Qiu[†], Lei Wang, Jianjun Liu, Shuangqiang Chen[*], Chilin Li[*]

Y. Zheng, Prof. C. Li

CAS Key Laboratory of Materials for Energy Conversion, Shanghai Institute of Ceramics, Chinese Academy of Sciences, Shanghai 201899, China. Email: chilinli@mail.sic.ac.cn

Y. Zheng, Dr. W. Qiu, Prof. J. Liu, Prof. C. Li

Center of Materials Science and Optoelectronics Engineering, University of Chinese Academy of Sciences, Beijing 100049, China.

L. Wang, Prof. S. Chen

Department of Chemical Engineering, School of Environmental and Chemical Engineering, Shanghai University, Shangda Road 99, Shanghai 200444, China.

Email: chensq@shu.edu.cn

Y. Zheng, Dr. W. Qiu, Prof. J. Liu, Prof. C. Li

State Key Laboratory of High Performance Ceramics and Superfine Microstructure, Shanghai Institute of Ceramics, Chinese Academy of Sciences, 585 He Shuo Road, Shanghai 201899, China.

[†] These authors contribute equally to this work.

Experimental Section

Materials and Reagents: $NbCl_5$ (Aladdin, 99.9%), $Co(NO_3)_2{\cdot}6H_2O$ (Aladdin, 99.99%), $Mn(NO_3)_2{\cdot}6H_2O$ (Aladdin, 98%), isopropanol (Aladdin, 99.5%), glycerol (Sinopharm, 99%), tris(hydroxymethyl)methyl aminomethane (Tris, Aladdin, 99.9%), HCl (Sinopharm, 36% - 38%), dopamine hydrochloride (Aladdin, 99.9%), anhydrous ethanol, deionized water.

Synthesis of Co-NbO: 0.5 mmol $NbCl_5$ and 0.05 mmol $Co(NO_3)_2{\cdot}6H_2O$ (or $Mn(NO_3)_2{\cdot}6H_2O$) were thoroughly dissolved in the isopropanol (30 ml) and ethanol (10 ml) solution with the assistance of agitation. 8 ml glycerol was added under vigorous stirring for 30 min to promise the homogeneous dispersion. Then the solution was transferred into a 50 ml Teflon-sealed autoclave and heated at 180 °C for 24 h in a drying oven. After naturally cooling down to room temperature, the obtained precipitation was washed with anhydrous ethanol/deionized water for three times and dried at 60 °C overnight. The dried powder was calcined at 600 °C in air for 3 h with a heat rate of 3 °C/min. Finally, the obtained light blue powder was denoted as Co-NbO (or Mn-NbO). In comparison, the pristine Nb_2O_5 powder was synthesized based on the same procedure without the use of cobalt precursor, denoted as P-NbO.

Synthesis of PDA coated Co-NbO: The obtained Co-NbO powder (200 mg) was dispersed in 200 mL 10 mM Tris/deionized water solution with violent agitation. The PH value was adjusted to 8.5 with 1 M HCl. 50 mg dopamine hydrochloride was added and stirred dramatically for 24 h to promise the conformal coating of polydopamine (PDA) on the surface of Co-NbO particles. The PDA coated Co-NbO particles were collected by vacuum filtration and washed with anhydrous ethanol for 3 times, followed by annealing at 450 °C in N_2 for 3 h with a heat rate of 3 °C/min. The obtained gray powder was denoted as PDA-Co-NbO.

Physical characterizations: The crystal structures and phase compositions of P-NbO, Co-NbO and PDA-Co-NbO were identified by X-ray powder diffractometer (XRD, Bruker, D8 Discover, Cu Kα) with 2θ ranging from 10° to 80°. The morphologies and microstructures were characterized by scanning electron microscopy (SEM, Magellan 400L, FEI, operated at 2.0 kV). Element distribution in PDA-Co-NbO powder was exhibited by using the energy dispersive X-ray spectrometer on SEM. Transition

electron microscopy (TEM, JEOL JSM-6700F, operated at 200 kV) was used to collect the common TEM images and high-resolution TEM (HRTEM) images of PDA-Co-NbO. The elemental composition and corresponding valence state on the surface of PDA-Co-NbO particles were analyzed by X-ray photoelectron spectroscopy (XPS, ESCAlab-250). Raman spectra were collected in the scope of 50-990 cm $^{-1}$ on a Renishaw InVia Reflex using a 532 nm laser as the excitation source. Electron paramagnetic resonance (EPR) spectra were collected by a JEOL-FA200 instrument with a magnetic field strength from 310 mT to 360 mT.

Measurements of electrochemical performance: For the rate and cycling performance tests, CR2032-type coin cells were assembled in argon-filled glovebox (with water and oxygen contents below 0.1 ppm) by taking P-NbO, Co-NbO or PDA-Co-NbO as working electrode and lithium foil as counter electrode. All three working electrodes were fabricated by blending active material powder, carbon black and poly(vinylidene fluoride) (PVDF, dissolved in 1-methyl-2-pyrrolidinone in a ratio of 1 mg/20 μL) with a weight ratio of 7:2:1. The uniform slurry dispersed by mortar and pestle was casted onto copper foil followed by drying in vacuum oven at 60 °C for 18 h. Then, the electrodes were cut into small disks with a diameter of 10 mm, with a mass loading of 1.5-1.8 mg/cm^2 if no special notation. 150 μL commercial electrolyte (Sigma-Aldrich) was employed, and it consists of 1 M lithium hexafluorophosphate ($LiPF_6$) dissolved in ethylene carbonate (EC) and diethyl carbonate (DEC) with a volume ratio of 1:1. Glass fiber (Whatman) was adopted as separator for the assembly of Li/P-NbO, Li/Co-NbO and Li/PDA-Co-NbO cells. Galvanostatic charge/discharge measurements were performed in the potential scope of 0.4-3.0 V versus Li^+/Li, under different current densities from 0.2 to 10 A/g (i.e. from 1 C to 50 C, 1 C = 200 mA/g) on Land multichannel battery testing system (CT2001A) at room temperature. For the assembly of full cells, the lithiated PDA-Co-NbO or Co-NbO electrode (precycled at 200 mA/g for 5 cycles and disassembled from Li/PDA-Co-NbO or Li/Co-NbO cells) and $LiNi_{0.8}Co_{0.1}Mn_{0.1}O_2$ or $LiFePO_4$ electrode (on aluminum foil as current collector) were adopted as anode and cathode, respectively. Galvanostatic intermittent titration technique (GITT) was

performed at a small current density of 20 mA/g for 1 h with subsequent static interval of 6 h. Electrochemical impedance spectroscopy (EIS) measurements of Li/P-NbO, Li/Co-NbO and Li/PDA-Co-NbO cells before cycling were performed with Solartron frequency analyzer (1260-1296), ranging from 1 MHz to 0.01 Hz. Cyclic voltammetry (CV) measurements were finished by using an electrochemical workstation (VersaSTAT3, AMETEK Scientific Instruments) in the potential scope of 0.4-3.0 V at stepwise increasing scan rates from 0.1 to 5 mV/s.

Simulation and calculation: Our calculations were performed using the plane wave basis Vienna ab initio simulation package (VASP),[1,2] implementing the generalized gradient approximation (GGA) of Perdew-Burke-Ernzerhof form.[3] The interaction between ions and electrons is described by the projector augmented wave (PAW) method,[4,5] with plane waves up to a cutoff energy of 600 eV. The Brillouin-zone integrations were performed on the grid of Monkhorst-Pack procedure.[6] The convergence of the total energy with respect to the kinetic energy cutoff and *k*-point sampling has been carefully examined.

Measurements of in situ XRD and in situ Raman: In situ XRD measurement of PDA-Co-NbO was performed by X-ray diffractometer (Bruker, D8 Discover, Cu Kα) in the scope of 20°-60° with a scan rate of 3 °C/min. XRD data were collected with a charge/discharge interval of 10 min. Corresponding charge/discharge process was operated with an electrochemical window of 0.4-3.0 V at a current density of 100 mA/g on Land test system (CT2001A). The electrode slurry was fabricated by mixing PDA-Co-NbO, carbon black and PVDF in a mass ratio of 7:2:1 and casted on Beryllium plate, with an average loading of 3 mg/cm^2. The same electrolyte (1 M $LiPF_6$ in EC/DEC with a volume ratio of 1:1) used in coin cells was adopted to ensure the identical conditions. In-situ Raman spectra was collected in the scope of 100-600 cm^{-1} on a Renishaw InVia Reflex (3 mW) using a 524 nm laser as excitation source and the corresponding electrochemical test conditions are the same as those of in-situ XRD measurement.

Reference

[1] G. Kresse, J. Furthmuller, *Phys. Rev. B* **1996**, *54*, 11169.

[2] G. Kresse, J. Furthmuller, *Comp. Mater. Sci.* **1996**, *6*, 15.

[3] J. P. Perdew, K. Burke, M. Ernzerhof, *Phys. Rev. Lett.* **1996**, *77*, 3865.

[4] G. Kresse, D. Joubert, *Phys. Rev. B* **1999**, *59*, 1758.

[5] P. E. Blöchl, *Phys. Rev. B* **1994**, *50*, 17953.

[6] H. J. Monkhorst, J. D. Pack, *Phys. Rev. B* **1976**, *13*, 5188.

Formula S1

$$I_p = 2.69 \times 10^5 n^{3/2} A (D_{Li})^{1/2} v^{1/2} \Delta C_0$$

I_p is the peak current, n refers to the number of electrons participating in reaction, A is equal to the electrode area immersed in electrolyte, D_{Li} is Li^+ diffusion coefficient in electrode, v is the sweep rate and ΔC_0 is the driving concentration difference of Li^+ in lattices before and after the corresponding electrochemical process.

Supplementary Figures

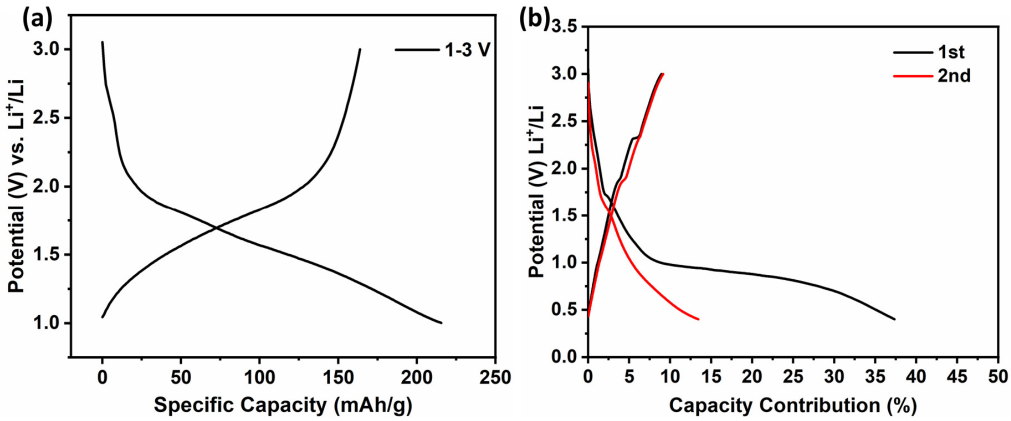

Figure S1. (a) Typical charge/discharge curves of T-Nb_2O_5 within a voltage range of 1-3 V. (b) Capacity contribution of carbon black in the 1st and 2nd cycles in T-Nb_2O_5 electrode within the voltage range of 0.4-3 V.

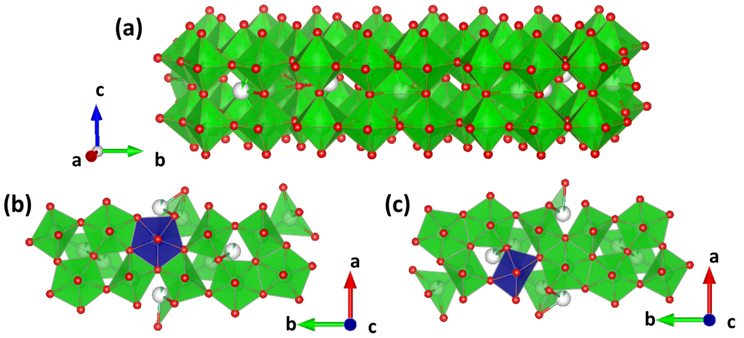

Figure S2. Crystallographic structures of (a) T-Nb_2O_5 and (b,c) Cocalt substituted T-Nb_2O_5 with NbO_n and CoO_n polyhedrons in green and blue respectively, Nb atoms in green, oxygen atoms in red, cobalt atoms in blue.

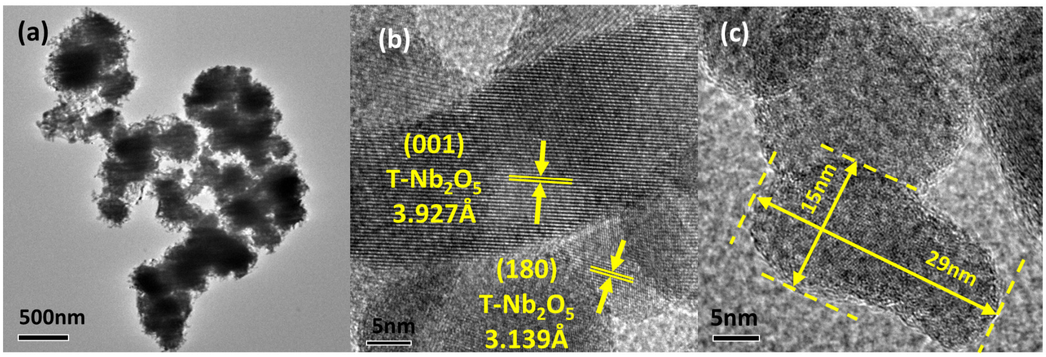

Figure S3. (a) TEM image and (b, c) HRTEM images of PDA-Co-NbO.

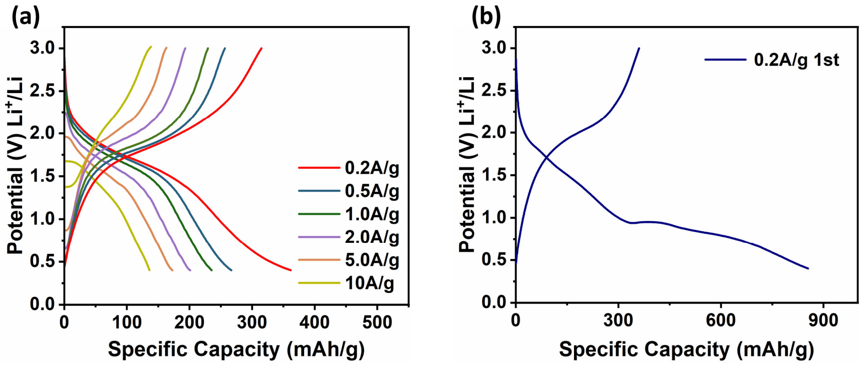

Figure S4. (a) Charge/discharge curves of PDA-Co-NbO at different current densities from 0.2 to 10 A/g in a potential scope of 0.4-3.0 V. (b) Charge/discharge curves of PDA-Co-NbO during the 1st cycle.

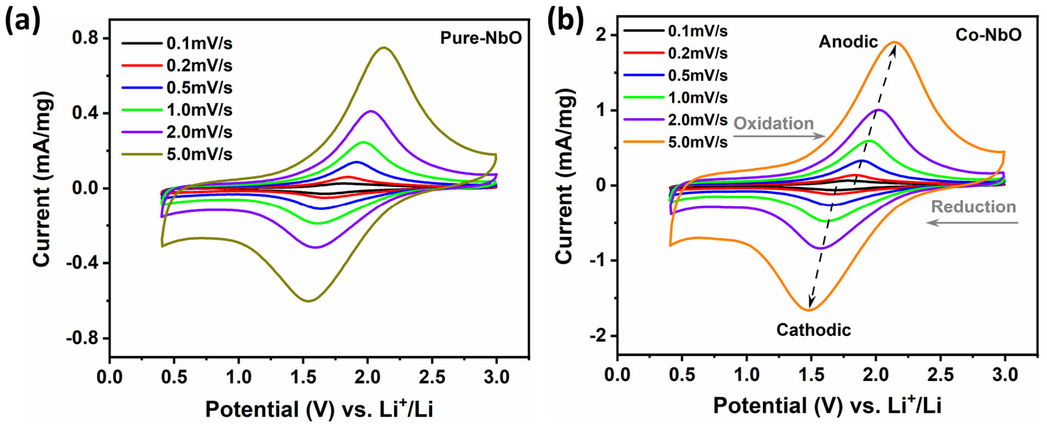

Figure S5. CV curves of (a) P-NbO and (b) Co-NbO at different scan rates from 0.1 mV/s to 5.0 mV/s.

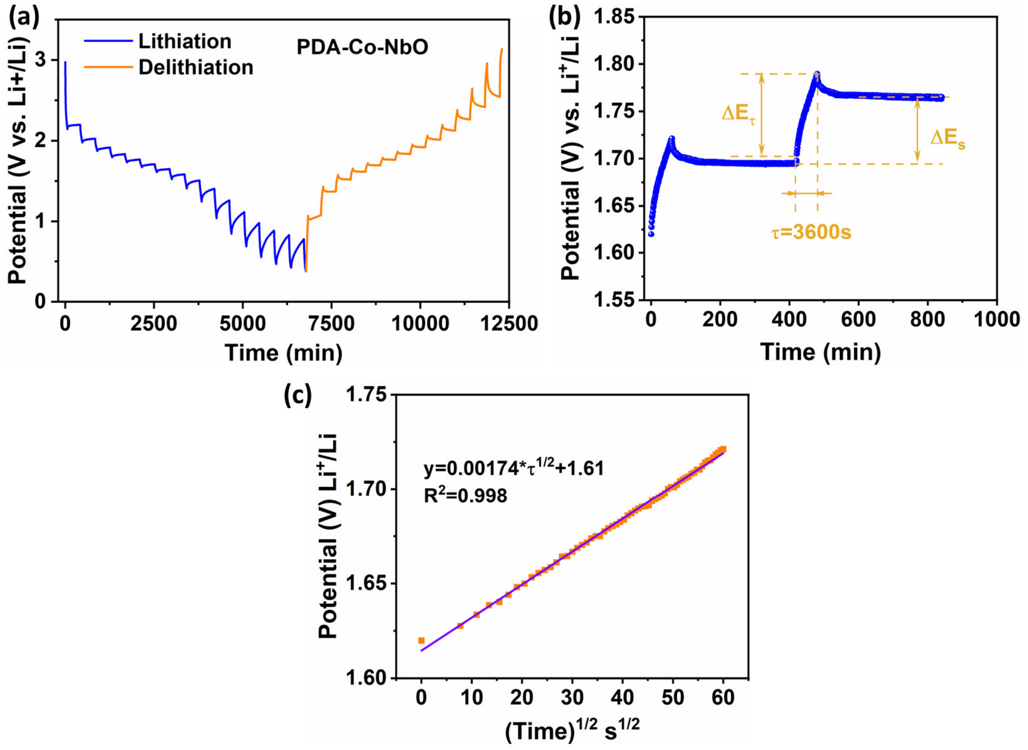

Figure S6. (a) GITT curves of PDA-Co-NbO during lithiation and delithiation. (b) Adjacent charging/standing voltage curves (versus time) for the interpretation of parameters in the calculation of Li^+ diffusion coefficient. (c) Plots of linear relationship between the square root of time and potential in the charge process of PDA-Co-NbO.

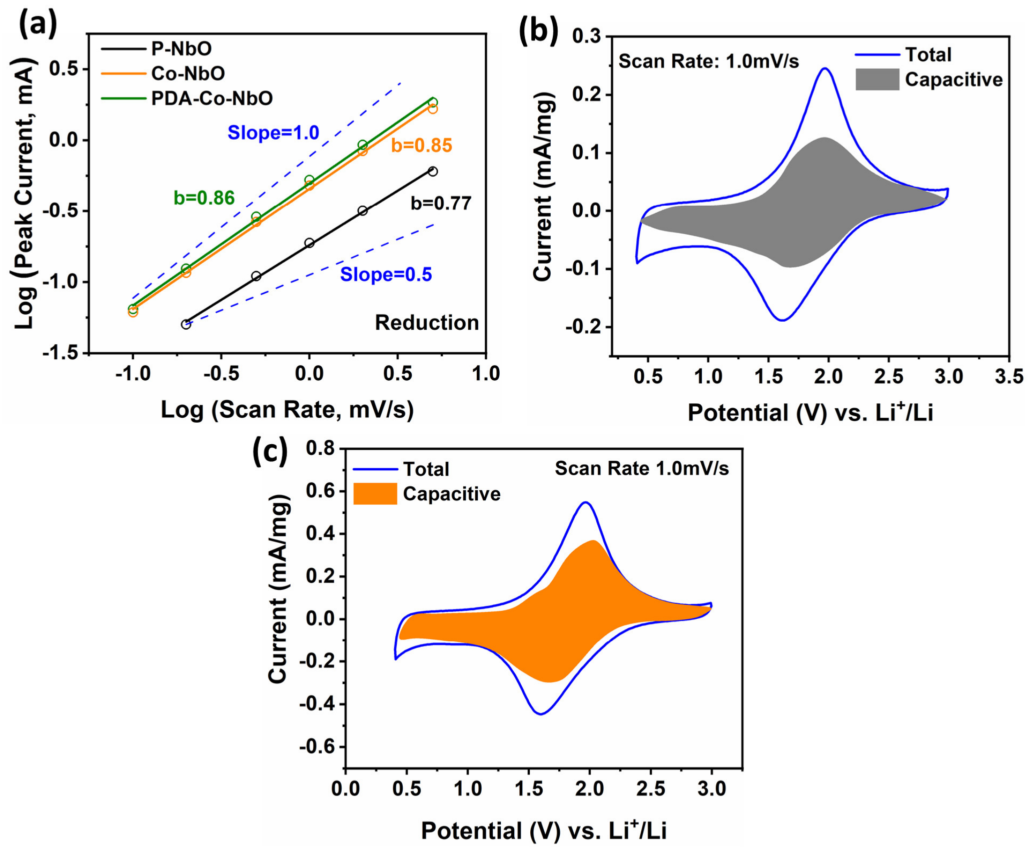

Figure S7. (a) b-value determination from the relationship between peak current and scan rate in cathodic process for P-NbO, Co-NbO and PDA-Co-NbO based on $\log i(V) = b \log v + \log a$. CV curves of (b) P-NbO and (c) Co-NbO at a scan rate of 1 mV/s with gray and orange areas assigned for pseudocapacitive current contribution estimated according to the formula of $i(V) = k_1 v + k_2 v^{1/2}$.

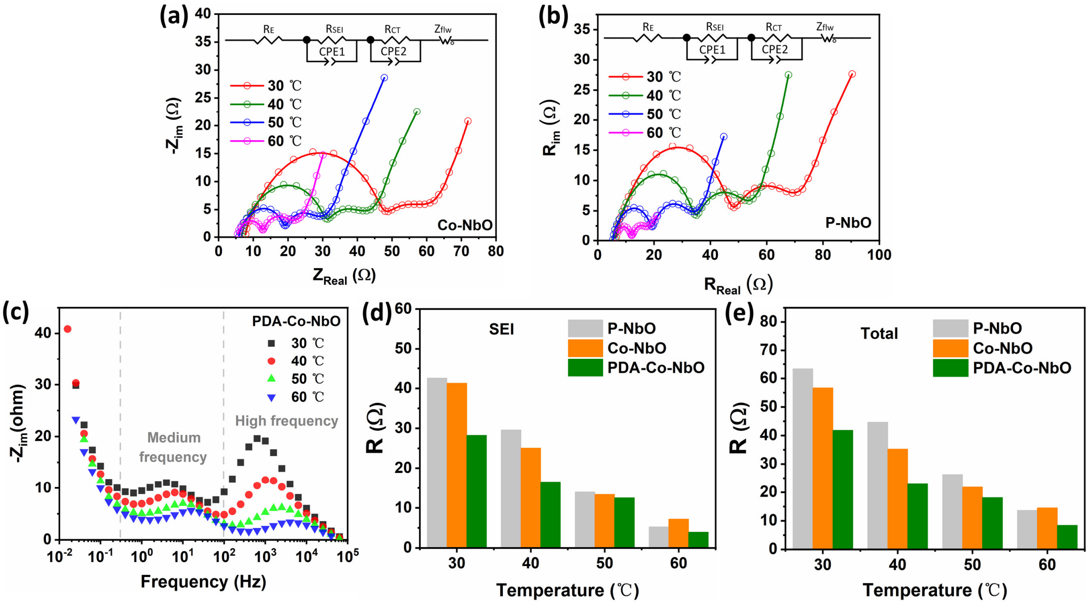

Figure S8. Nyquist plots of (a) P-NbO and (b) Co-NbO based cells at different temperatures fitted with the equivalent circuit in the inset. (c) Scatter plots of imaginary impedance as a function of frequency ranging from 0.1 MHz to 0.01 Hz at different temperatures for PDA-Co-NbO. Column graphs of (d) SEI resistance and (e) total resistance (R_{SEI}+R_{CT}) at different temperatures for P-NbO, Co-NbO and PDA-Co-NbO.

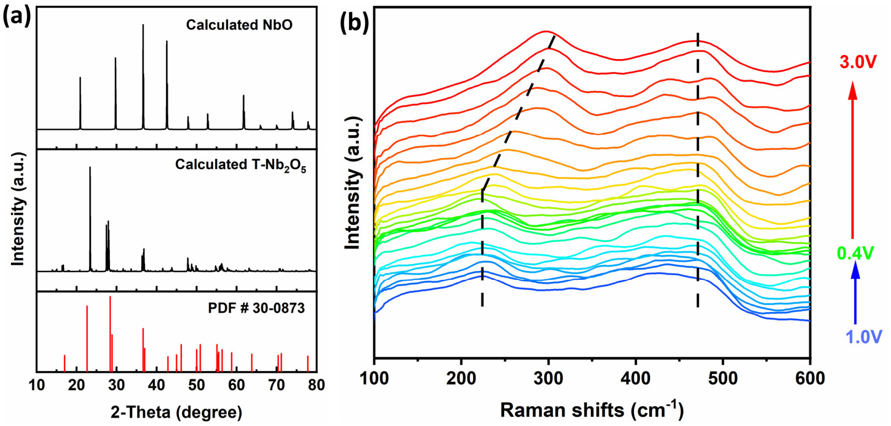

Figure S9. (a) Calculated XRD patterns of NbO and T-Nb_2O_5 structures from PDOS analysis. (b) In situ Raman curves of PDA-Co-NbO during the second charge/discharge process.

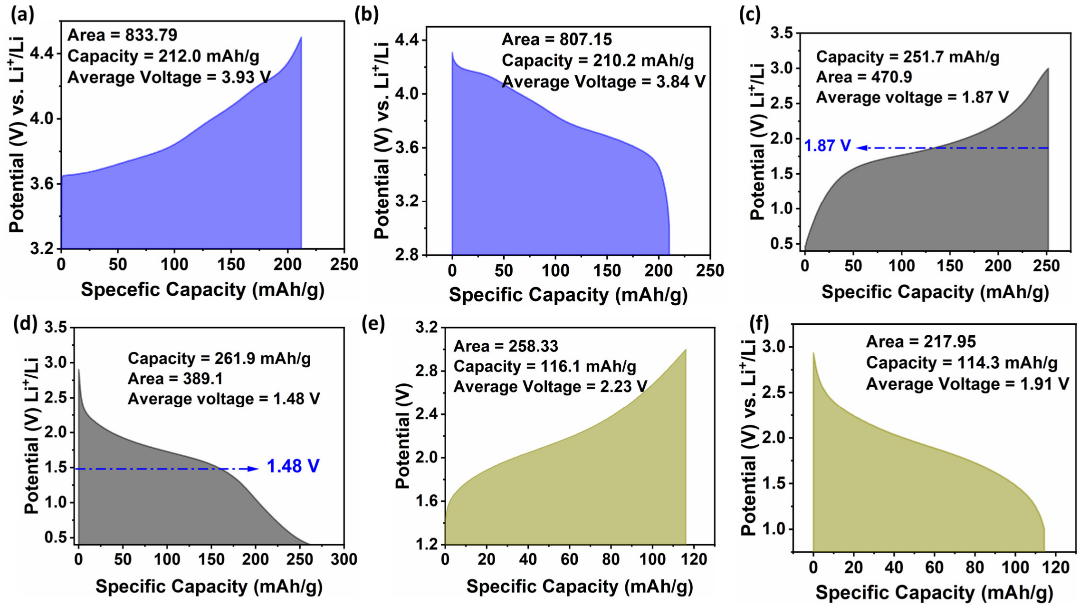

Figure S10. Average voltage estimation from the (a) charge and (b) discharge profiles of NCM811 cathode. Average voltage estimation from the (c) charge and (d) discharge profiles of Co-NbO anode. Average voltage estimation from the (e) charge and (f) discharge profiles of Co-NbO/NCM811 full cell.

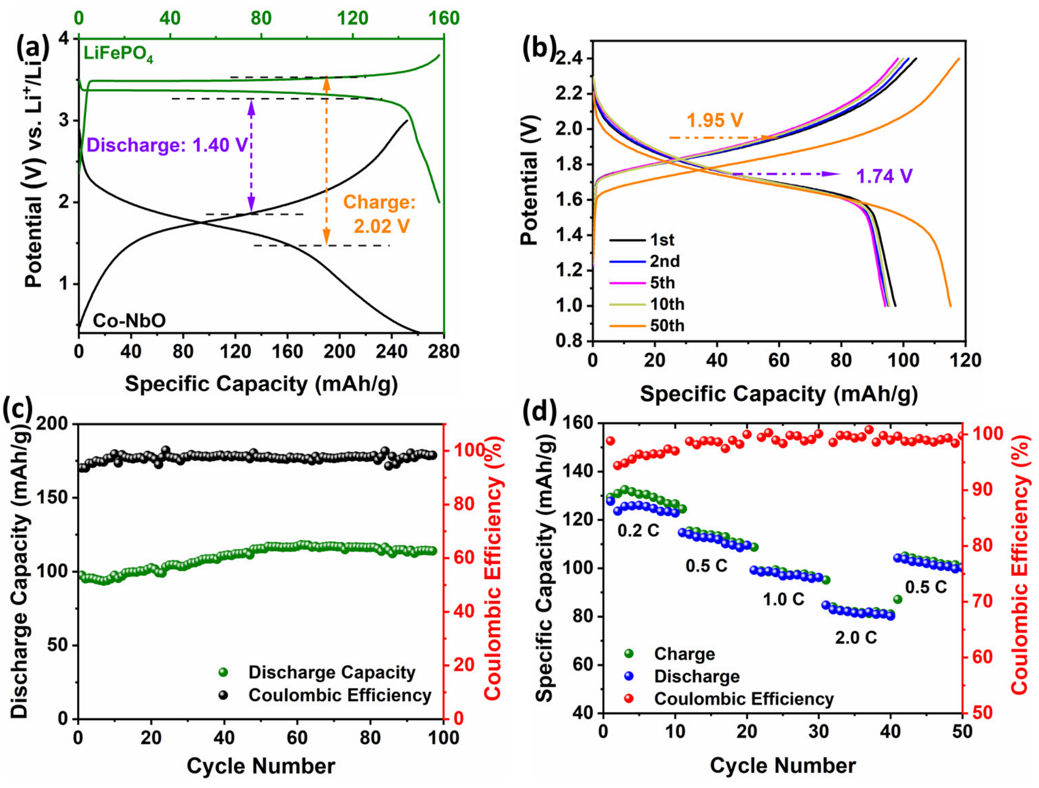

Figure S11. (a) Charge/discharge voltage profiles of Co-NbO anode at 200 mA/g and $LiFePO_4$ cathode at 170 mA/g. (b) Charge/discharge curves of Co-NbO/$LiFePO_4$ full cell at different cycling stages. (c) Cycling performance of Co-NbO/$LiFePO_4$ full cell at 0.2 C (1 C = 170 mA/g). (d) Rate performance of Co-NbO/$LiFePO_4$ full cell at different rates from 0.2 C to 2 C.

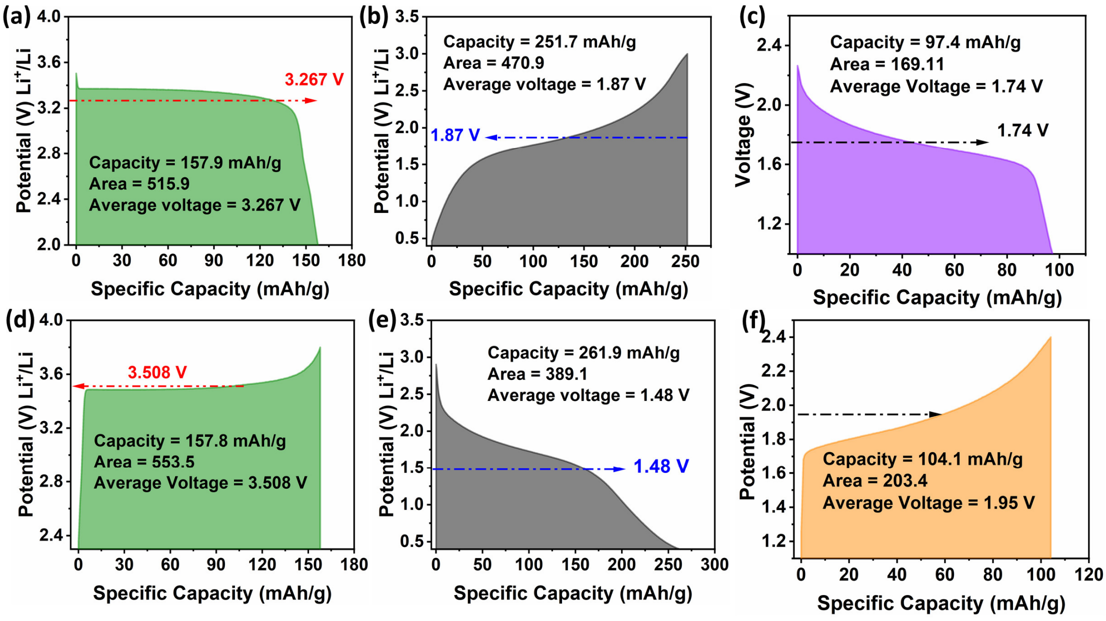

Figure S12. Average voltage estimation from the (a) discharge profile of $LiFePO_4$ cathode, (b) charge profile of Co-NbO anode and (c) discharge profile of Co-NbO/$LiFePO_4$ full cell. Average voltage estimation from the (d) charge profile of $LiFePO_4$ cathode, (e) discharge profile of Co-NbO anode and (f) charge profile of Co-NbO/$LiFePO_4$ full cell.
